# Missing Inferior Vena Cava on POCUS: A Case of Left-Sided IVC with Azygos Continuation 

**DOI:** 10.24908/pocus.v8i1.16240

**Published:** 2023-04-26

**Authors:** Ankit Mehta, Kreegan Reierson, Benji Mathews

**Affiliations:** 1 Department of Internal Medicine, HealthPartners St. Paul, MN USA; 2 Department of Internal Medicine, University of Minnesota Minneapolis, MN USA

**Keywords:** Diagnostic pitfalls, Inferior Vena Cava, POCUS

## Abstract

The merits of utilizing point of care ultrasound (POCUS) in acutely ill patients is leading to a widespread embrace. Assessment of IVC via POCUS as part of a comprehensive multi-organ approach can help guide volume tolerance. Anatomical/developmental variations of IVC can vary widely in prevalence. As the use of POCUS expands as a diagnostic modality, it is prudent for frontline POCUS users to be cognizant of the IVC anomalies. We present a case of left sided IVC with azygous continuation discovered with POCUS that was performed to assess the volume status of the patient. This case illustrates that the awareness of different anomalies of the IVC is necessary for POCUS users to prevent misinterpretation of aberrant vessels and avoid diagnostic pitfalls.

## Introduction

Point of care ultrasound (POCUS) is a crucial diagnostic tool widely considered a standard modality to manage acutely ill patients. Use of POCUS has expanded dramatically in the last decade. Evidence suggests that POCUS leads to timely diagnosis, improved outcomes and higher patient satisfaction 1, 2. Its portability, low cost compared to consultative imaging and real time application allows clinicians to quickly and effectively manage hemodynamically unstable patients. Assessment of respiratory variation of the inferior vena cava (IVC) via POCUS as part of a broader exam can help guide volume tolerance 3, 4. POCUS IVC evaluation is therefore a useful tool to accurately gauge the right atrial pressure and get at the volume status of patients.

Anatomical/developmental variations of IVC have been reported to have prevalence anywhere between 0.07 to 8% in general population 5, 6. Most IVC anomalies are discovered incidentally, remain silent and rarely manifest clinically with a symptomatic pathology. As the use of POCUS widens, presence of these anatomical variants of IVC can lead to potential misdiagnosis, and pose challenges with therapeutic interventions. In the existing radiology literature, anomalous IVC has been associated with increased risk of DVT, can be misdiagnosed as tumors or lymphadenopathy, and has potential implications with surgical or radiologic interventions 5, 7. No such reports exist on anomalous IVC causing misdiagnosis with POCUS. With POCUS use becoming a standard of care, awareness of these developmental anomalies by frontline clinicians is crucial.

An isolated left-sided IVC has a prevalence of 0.2 to 0.5% 8and has been known since 1793 incidentally noted on cadaveric dissections. Advances in medicine now allow clinicians to image patients at the bedside. We present a case of left sided IVC discovered with POCUS hitherto not reported in the literature.

## Case report

60-year-old woman with history of chronic lower extremity venous insufficiency and ulcerations with recurrent cellulitis, hypothyroidism, anxiety and degenerative joint disease of the spine presented with leg pain, progressive weakness, subjective fevers, and confusion. She was noted to have hypothermia, lactic acidosis, acute kidney injury and several metabolic derangements along with hypotension that progressed to septic shock despite supportive cares and broad-spectrum antibiotics. Initially she required two vasopressors along with aggressive fluid resuscitation. Her focal septic source was deemed to be lower extremity cellulitis with acute on chronic ulcerations. During the course of her illness, she developed diarrhea and abdominal pain and was diagnosed with Clostridium difficile colitis, treated with oral vancomycin.

**Figure 1:**
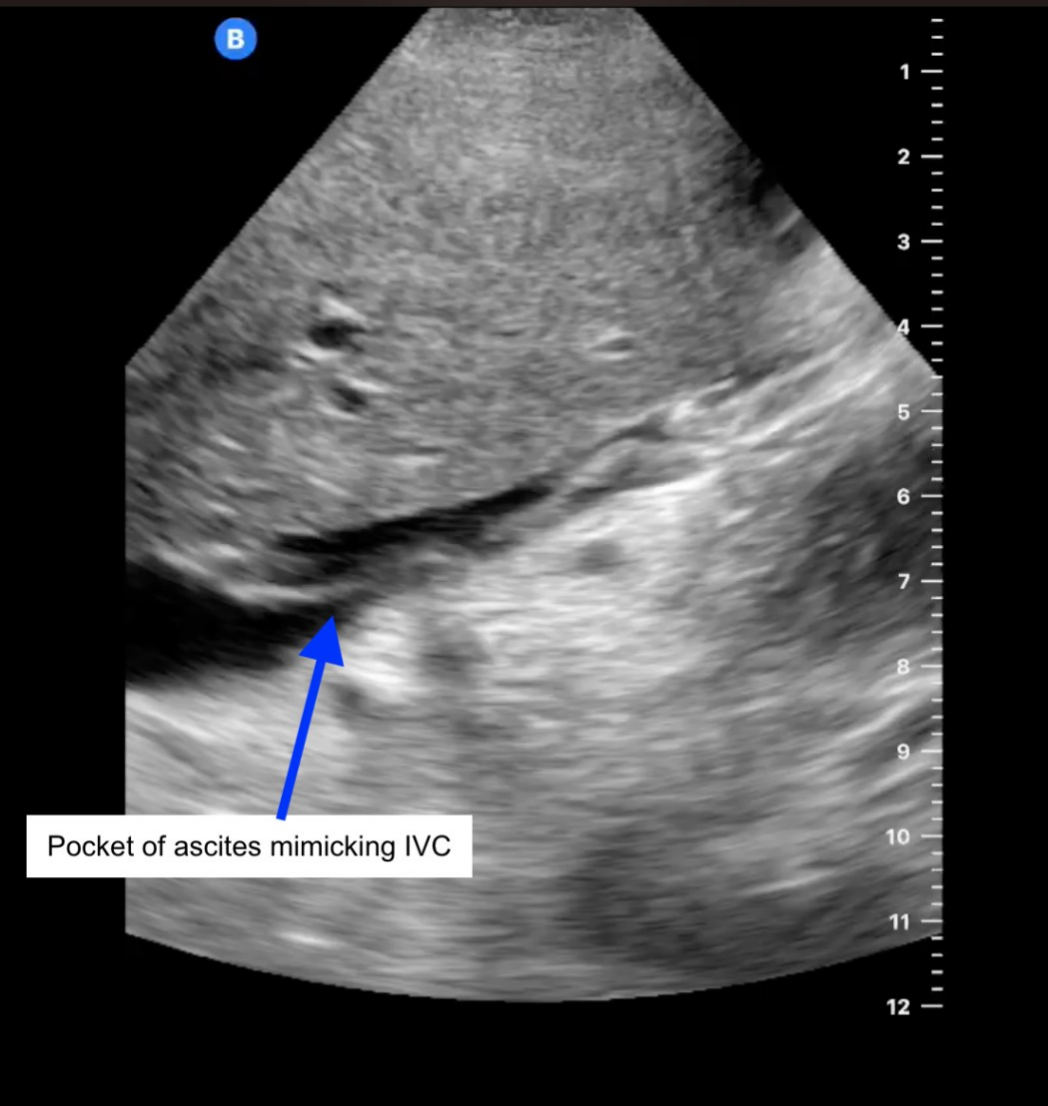
Ascites pocket with stranding – initial mimic for IVC raising concern for IVC thrombus. Color Doppler ruled out presence of vascular structure. (POCUS subcostal view in longitudinal axis. Probe is in subcostal position with marker pointing cranially).

**Figure 2:**
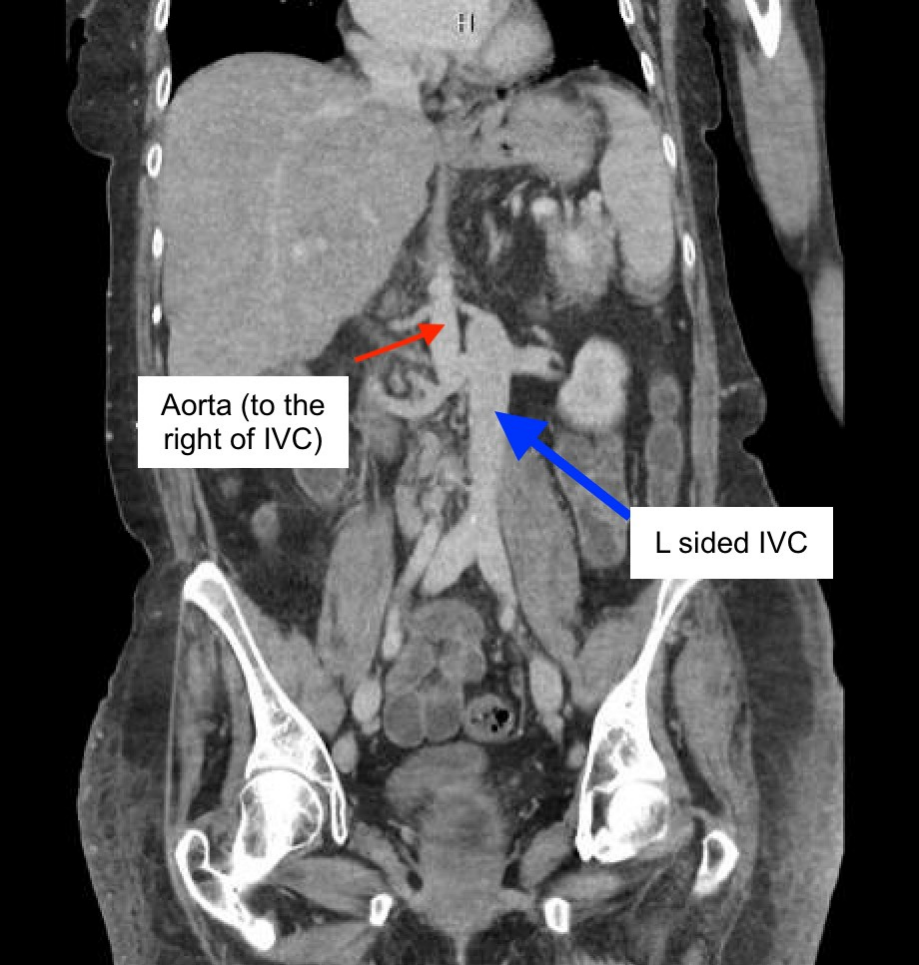
L sided IVC (blue arrow) with bilateral renal vein insertion. Aorta on the R side (red arrow). (CT abdomen/pelvis w/contrast - coronal view).

**Figure 3:**
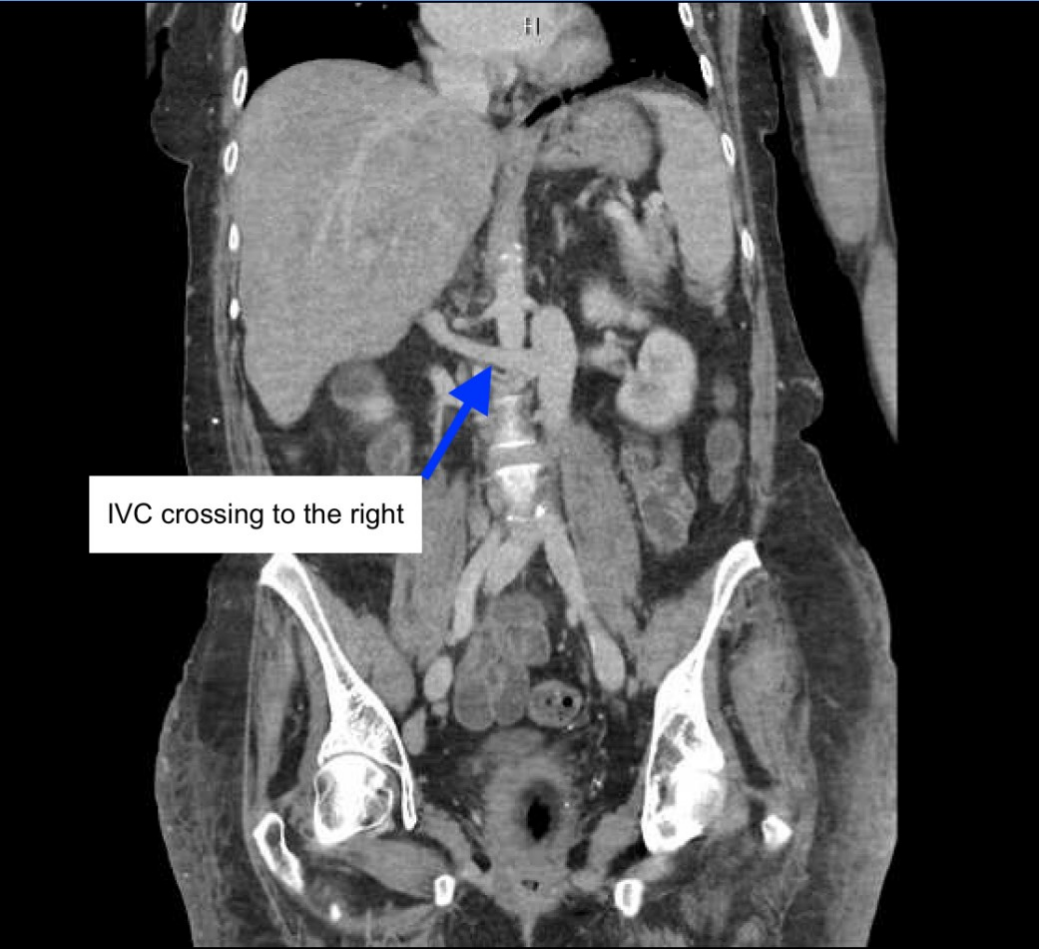
L sided IVC crossing to the right anterior to the aorta. (CT abdomen/pelvis w/contrast - coronal view).

**Figure 4:**
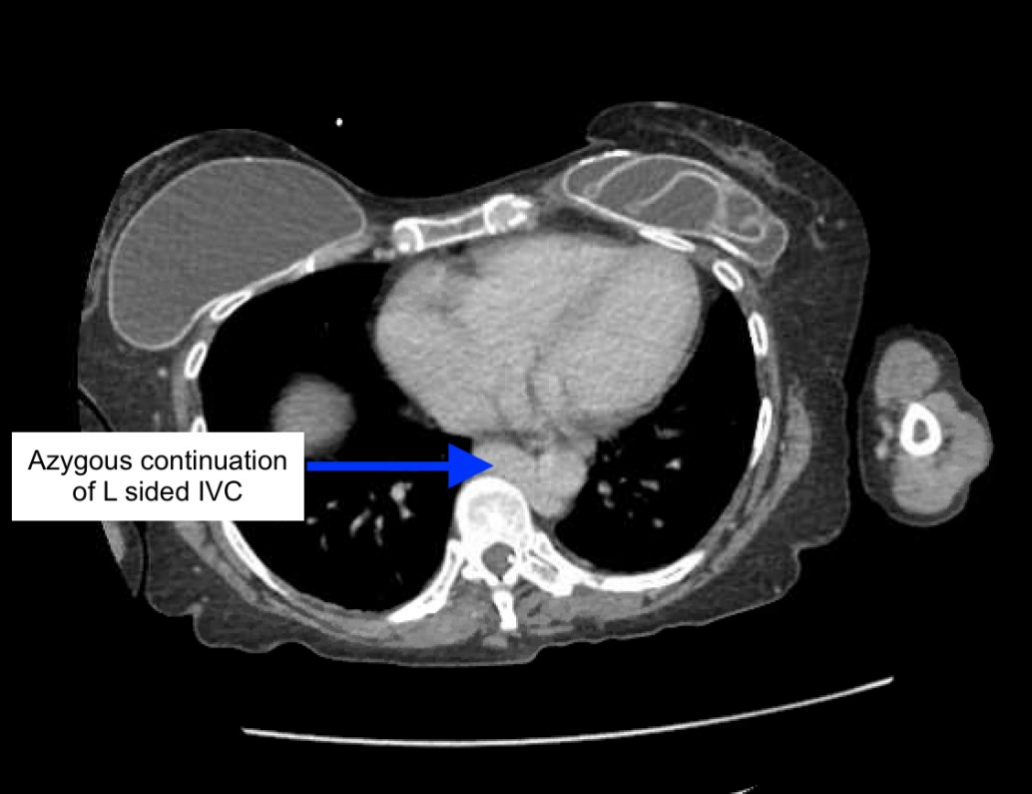
Azygos continuation of L sided IVC (blue arrow). (CT scan abdomen/pelvis w/contrast - axial plane).

**Figure 5:**
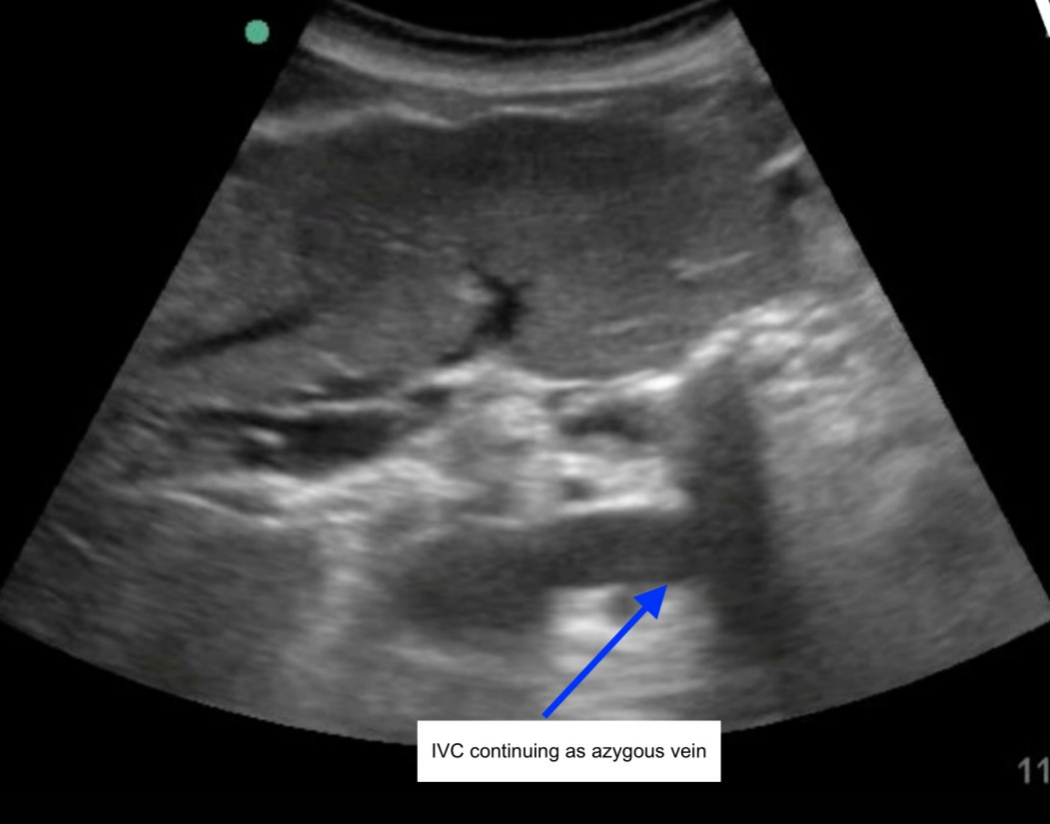
POCUS view of IVC continuing as azygos (blue arrow). (Subcostal view – image obtained by following IVC in long axis (near the sagittal plane), and then rotating the probe in a counter-clockwise fashion along the long axis of the individual diverging vessels, settling on the azygos vein along its specific long axis. Thus, the image is oriented with the dot aligning along a plane between the transverse plane and the sagittal plane).

Her course in the hospital was prolonged (over 21 days) and complex. As her critical illness resolved, she was noted to have marked anasarca with ongoing sinus tachycardia, that worsened with diuretics. POCUS was performed to assess her volume status. POCUS IVC in the longitudinal axis showed anechoic linear tubular structure with mobile hyperechoic density (Figure 1, Video S1) initially raising suspicion for IVC thrombus. Use of color Doppler ruled out this anechoic structure as a vessel. Additional abdominal scanning suggested small fluid pockets raising suspicion for ascites. Anomalous IVC was suspected and explored further with CT abdomen/pelvis that showed L sided IVC with azygous continuation superior to the level of renal veins (Figure 2-5, Video S2).

## Embryogenesis

**Figure 6:**
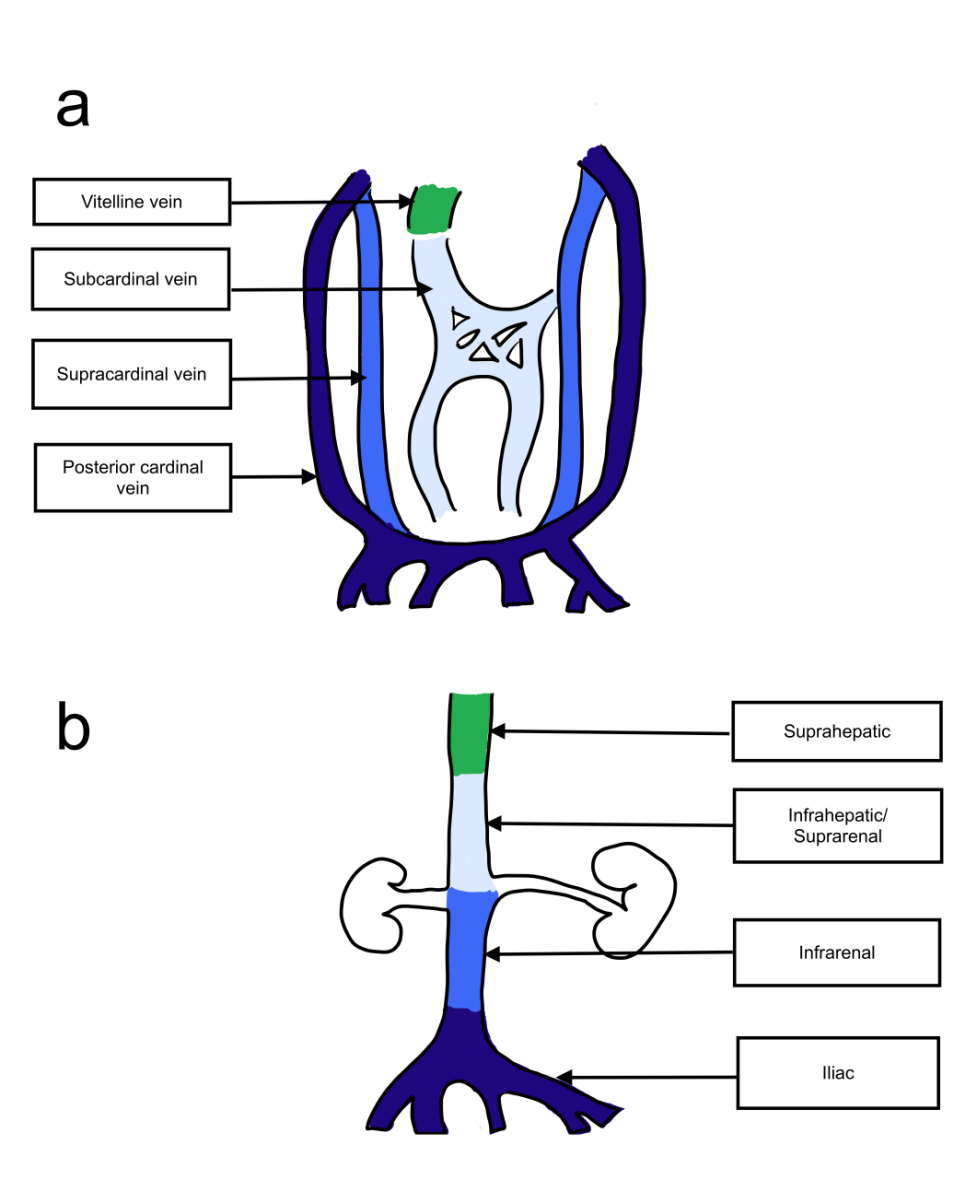
IVC embryogenesis. a) Primitive veins contributing to the development of IVC. b) Adult IVC with its originating venous systems.Suprahepatic segment originates from the vitelline veins. Suprarenal/infrarenal segment originates from the subcardinal veins. Infrarenal segment from the supracardinal veins. Iliac veins from the posterior cardinal veins.

A normal mature IVC has 4 segments: suprahepatic, suprarenal/infrahepatic, infrarenal and the iliac veins confluence. Development of IVC involves a precise choreography that leads to the normal configuration of right-sided IVC in most adults. This process occurs during 4-8^th^ week of gestation. The embryonic veins contributing to the formation of the IVC are the vitelline and the cardinal veins. Cardinal veins have a predominant role in contributing to the formation of IVC. There are three pairs of cardinal veins: supracardinal, posterior cardinal and subcardinal. Figure 6 illustrates the primitive venous system and their contribution in the formation of mature IVC segments.

The primitive three pairs of cardinal veins are symmetric but following successive anastomosis, crossings and fusions turn into a single large trunk on the right side whereas the left sided vessels slowly atrophy to the point of obliteration (Figure 6a). Given the complicated nature of this metamorphosis that involves selective progression and regression of various vessels to form the final IVC, there are many opportunities for the abnormal developments that can lead to anatomical variations of IVC.

A left sided IVC results from the persistence of left supracardinal veins as the right suprarenal vein regresses. An isolated left sided IVC ascends on the left and has multiple possible pathways to drain into right atrium. Firstly, the IVC ascending on the left crosses the midline anterior to the aorta after receiving both renal veins and continues the course on the right, eventually draining into the right atrium. Secondly, after crossing the midline, IVC gets interrupted and continues as azygos (in the reported case) continuing into SVC and eventually draining into the right atrium. Lastly (and least commonly of the three routes) left sided IVC continues caudally on the left as hemiazygos. Azygos and hemiazygos continuation of IVC is prevalent up to 0.6% of the population 5.

## Discussion

POCUS is widely deemed the standard of care for managing acutely ill patients. As the use of POCUS expands as a diagnostic modality, it is prudent for frontline POCUS users to be cognizant of the IVC anomalies. Although these venous variations can vary widely in prevalence, awareness of the different anomalies of the IVC is necessary for POCUS users to prevent misinterpretation of aberrant vessels and avoid diagnostic pitfalls. 

As presented in the case, the patient had an isolated left sided IVC with azygos continuation. Left sided IVC has been associated with increased prevalence of venous thromboembolism in lower extremities and venous insufficiency 9. It is considered that the collaterals that physiologically compensate for venous return likely increase the risk of DVTs. Our patient has history of venous insufficiency and chronic leg wounds leading to recurrent infections and sepsis.

The left sided IVC in this case continued as azygos eventually joining the right atrium. Azygos continuation of IVC has been associated with complex maladaptive syndromes including congenital heart defects, polysplenia, and situs anomalies 10.Additionally, azygos dilation due to its discharging function, can lead to right sided mediastinal widening on imaging 11.Our patient did not have any evidence of these associated syndromes.

IVC anomalies are well documented in existing radiology literature, including left sided IVC. However, discovery of left sided IVC with azygos continuation by POCUS has not been reported ever before. In addition to this rare finding, this case was particularly challenging with presence of ascites initially mimicking IVC at its usual anatomical landmarks. This case also underscores the basic principles of using POCUS to ensure a pathology should be evaluated in different planes and a potential vascular structure should be confirmed using color Doppler to avoid pitfalls. Table 1 summarizes the tips for acquiring IVC and avoiding pitfalls. 

**Table 1:** Tips for acquiring POCUS IVC images and avoiding pitfalls.

Tips for acquiring POCUS IVC images and avoiding pitfalls
Optimize patient positioning and ergonomics for scanning. Have knees bent, if possible, relax abdominal musculature. Use patient’s breathing to aid in image acquisition (deep breathing, inspiratory hold if possible).
When it is difficult to identify IVC, start with a subcostal 4-chamber view focusing on the right atrium and then rotate the transducer counterclockwise, to obtain IVC view in longitudinal axis at the IVC-RA junction.
An ideal long-axis view of the IVC shows the IVC entering the right atrium and a segment of the hepatic vein joining the IVC. Ensuring both landmarks being visible: RA-IVC junction and hepatic vein helps avoid mistaking the IVC for aorta.
It is imperative to visualize the IVC longitudinally with the transducer centered on the long axis to assess the true diameter accurately and avoid “cylinder” effect.
When encountering any pathology, rule of thumb for POCUS is to visualize the structure in longitudinal and transverse axis. Adding color Doppler helps differentiating a vascular structure from a non-vascular one.
When the subxiphoid view is not obtainable (surgical dressings etc.), a longitudinal IVC view maybe acquired through a right lateral transabdominal coronal approach. The transducer is placed in the right anterior to mid axillary line with the orientation marker towards the patient’s head. The IVC can be seen adjacent to the liver and the aorta is often seen parallel to it in the far field of the image.

It is equally important to be mindful of the errors in incorporating POCUS IVC use in patient care. Traditionally IVC has been used for the assessment of the volume status; however exclusively relying on IVC diameter and collapsibility has been suboptimal in identifying volume status 12. Though the IVC diameter and its dynamics during respiratory cycles may allow a rough estimation of right atrial pressure, there are significant pitfalls and gray-zone conditions that alter the relationship between size of vein and actual volume status. Therefore, it is essential to integrate IVC analysis with a comprehensive multi-organ approach. Additionally, common pitfalls may even trick expert operators. Avoiding lateral translation (can erroneously give the impression of respiratory collapse) and misidentifying aorta as IVC (can erroneously give the impression of no respiratory variation) are important during IVC assessment.

In conclusion, as the merits of utilizing POCUS in critically ill patients leads to a widespread embrace, diagnostic pitfalls from IVC aberrancies should be recognized and factored in management.

## Consent/Ethics

The patient gave informed consent for this case report.

## Conflicts of Interest

The authors have no conflicts of interest to declare.

## Supplementary Material

Video S1 Ascites pocket with stranding – initial mimic for IVC raising concern for IVC thrombus.

Video S2 POCUS view of IVC continuing as azygos (blue arrow).
